# Partial ALPPS versus complete ALPPS for staged hepatectomy

**DOI:** 10.1186/s12876-019-1090-1

**Published:** 2019-10-26

**Authors:** Xukun Wu, Jiawei Rao, Xiaozhuan Zhou, Ronghai Deng, Yi Ma

**Affiliations:** Department of Organ Transplantation, First Affiliated Hospital of Sun, Yat-sen University, No. 58 Zhongshan 2nd Road, Guangzhou, 510080 China

**Keywords:** Partial ALPPS, Complete ALPPS, Future liver remnant, Hepatectomy

## Abstract

**Background:**

Associating liver partition and portal vein ligation for staged hepatectomy (ALPPS) can induce a stronger regenerative ability than traditional 2-stage hepatectomy (TSH). ALPPS has become popular for achieving fast hypertrophy in patients with an insufficient future liver remnant (FLR). However, ALPPS is associated with high morbidity and mortality. Partial ALPPS is a variation that may decrease the morbidity and mortality. The purpose of this study was to perform a meta-analysis comparing outcomes of ALLPS and partial ALLPS.

**Methods:**

PubMed, Embase, and Cochrane Library databases were searched for studies comparing partial ALPPS and complete ALPPS up to April 2019. Included studies were assessed by the Newcastle-Ottawa Scale (NOS). Weighted mean difference (WMD)/standard mean difference (SMD) and odds ratios (OR) with 95% confidence intervals (CIs) were calculated to compare FLR, time interval between stages, postoperative complications, and mortality between partial and complete ALPPS.

**Results:**

Four studies including 124 patients were included. FLR hypertrophy of partial ALPPS was comparable to complete ALPPS (*p* = 0.09). The time interval between stages was not different between the 2 procedures (*p* = 0.57). The postoperative complications rate of partial ALPPS was significantly lower than that of complete ALPPS (OR = 0.38; *p* = 0.03). The mortality rate of partial ALLPS (4.9%) was lower than that of complete ALLPS (18.9%), but the difference was not significant (OR = 0.37; *p* = 0.12).

**Conclusions:**

Partial ALLPS is associated with similar FLR hypertrophy and time interval between stages as complete ALLPS, and a lower complication rate. Further studies are needed to examine patient selection and outcomes of the 2 procedures.

## Background

Hepatectomy is the most effective treatment for large and/or multiple liver tumors [1]. However, an extensive hepatectomy cannot be performed if there will be an insufficient future liver remnant (FLR) because it may lead to post-hepatectomy liver failure (PHLF). To minimized PHLF, 20 to 25% of liver is needed in healthy livers while 30–35% in diseased livers [2]. In traditional 2-stage hepatectomy, liver hypertrophy can be induced after stage 1 by portal vein embolization (PVE) or portal vein ligation (PVL), and thus the FLR can meet the size requirement for the second stage procedure [3]. However, liver regeneration is slow making the time interval between the 2 stages long [4].

In 2007, Schlitt et al. performed the first “in-situ split” procedure [5], which was then named “Associating Liver Partition and Portal vein ligation for Staged hepatectomy” (ALPPS) by Clavien. ALPPS was performed by separating the future liver remnant and the diseased hemi-liver in the first stage with an in-situ split, in combination with PVL. Schnitzbauer et al. reported that ALPPS could induce rapid hypertrophy of the FLR (median FLR hypertrophy of 74% in 9 days) [5]. However, a serious complication (Clavien-Dindo > grade 3) rate of 44% and a mortality rate of 12% has limited the application of ALPPS [5].

Since the creation of the ALPPS Registry and the adoption of more stringent patient selection criteria, the overall mortality rate has dropped to 9% and serious complication rate to 27% [6]. Patients younger than 60 years old and those with colorectal cancer liver metastases (CRLM) have lower morbidity and mortality rates when they undergo ALPPS, while the prognosis for patients with gallbladder cancer or cholangiocarcinoma is poor. Technical modifications of ALPPS, such as partial ALPPS, tourniquet ALPPS, radiofrequency and microwave ALPPS, and mini-ALPPS have also been shown to be associated with reduced morbidity and mortality [7]. Partial ALPPS was described by Alvarez et al. [8], and consists of dividing the portal vein of the diseased hemi-liver up to middle hepatic vein. Compared with complete ALPPS, partial ALPPS induces comparable FLR hypertrophy with a lower morbidity rate and near zero mortality rate [8, 9]. However, Chan et al. have argued that complete ALPPS induces more rapid FLR hypertrophy than partial ALPPS, while not affecting the perioperative risk in chronic liver diseases [10]. As such, it is difficult to draw a firm conclusion due to the limited power of individual studies.

Thus, the purpose of this study was to conduct a meta-analysis comparing partial ALPPS with complete ALPPS with respect to FLR hypertrophy, the time interval between stages, postoperative complications, and mortality.

## Methods

### Study design and search strategy

PubMed, Embase, and Cochrane Library databases were searched up to April 2019, using the terms: “partial ALPPS” OR “p-ALPPS” OR “partial associating liver partition and portal vein ligation for staged hepatectomy”. We included studies without language or year restrictions, and the reference lists of all relevant studies were reviewed for additional potentially relevant studies.

### Inclusion and exclusion criteria

Studies were included when they met the following criteria: (1) Described the complete or partial ALPPS technique; (2) Were aimed at comparing FLR hypertrophy, time interval between stages, postoperative complication, and/or mortality. The excluded criteria were: (1) Studies using an animal model; (2) Non-comparative studies, review articles, letters, case reports, or journal editorials.

### Data extraction

Information extracted from the studies included author names, year of publication, number of patients, patient demographic data, indications for surgery, amount of parenchymal transection, FLR hypertrophy, time interval between stages, postoperative complications, and mortality rate. Two investigators extracted the data independently. A third investigator participated in when disagreements existed. We attempted to contact the study authors to obtain original data that could not be extracted from an article, but did not receive any reply.

### Quality assessment and statistical analysis

The modified Newcastle-Ottawa Scale was used to assess the quality of included studies. Data were collected following the Quality of Reporting of Meta-Analyses statement. Publication bias was assessed by the construction and visual inspection of funnel plots. Statistical analysis was performed according to instructions in the Cochrane Reviewer’s Handbook.

Weighted mean difference (WMD)/standard mean difference (SMD) and odds ratio (OR) and 95% confidence interval (CI) and a fixed-effects model was used to compare continuous and dichotomous variables. Values of *P* < 0.05 were considered to indicate a statistically significant difference. When the mean and standard deviation (SD) were not reported, median and range values were used to calculate the mean and SD with the formulas reported by Wan et al. [11] and Luo et al. [12]. All statistical analyses were performed using Review Manager version 5.2 for Windows (Cochrane Collaboration, Oxford, UK). Statistical heterogeneity was evaluated with a forest plot, chi-square test. and *I*^*2*^ statistic. If heterogeneity was observed, a random-effects model of analysis was used; otherwise a fixed-effects model was employed.

## Results

### Literature search

**Fig. 1:**
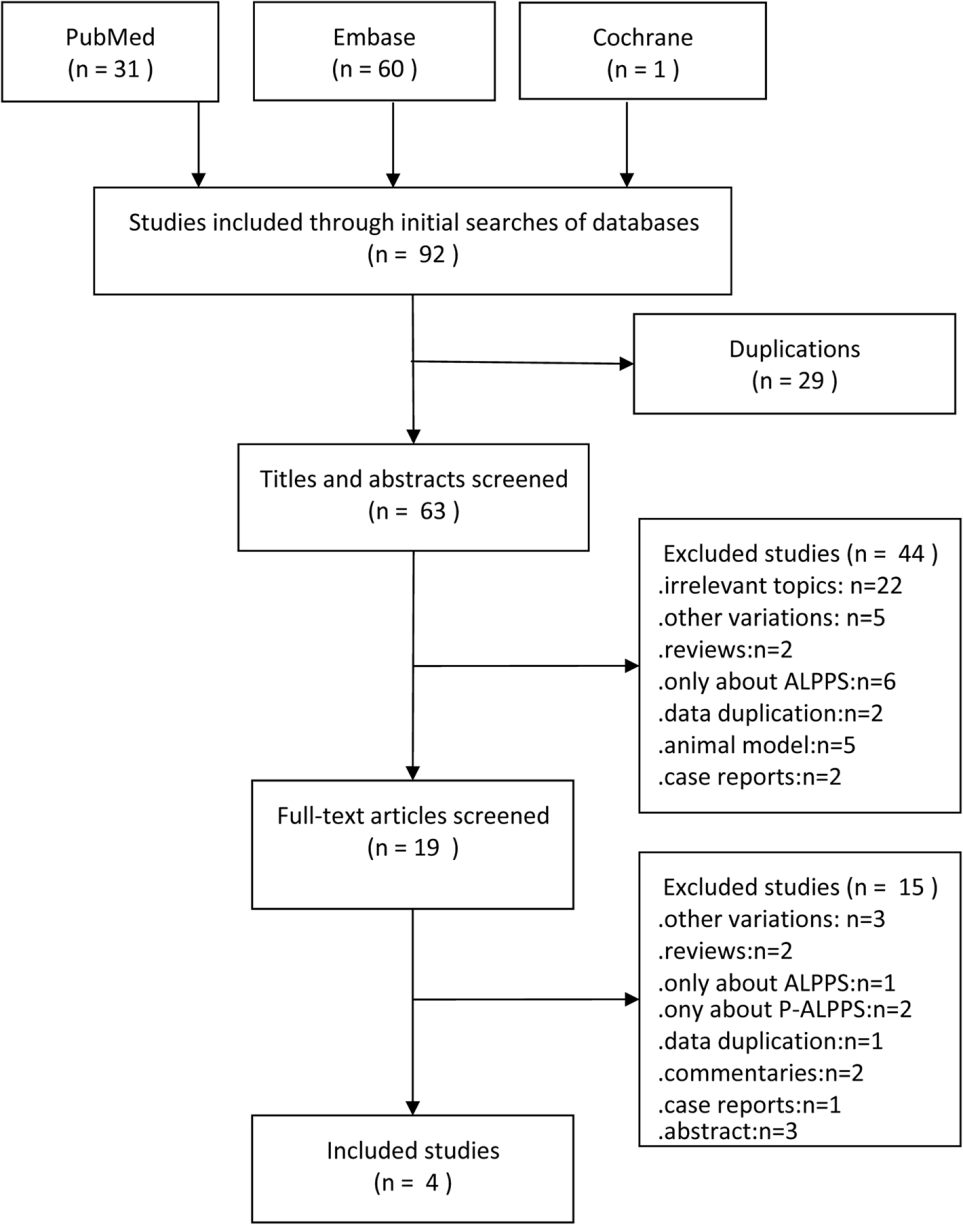
Flow diagram of study selection

A flow diagram of the literature search results, including the reasons for excluding studies, is shown in Fig. 1. Ninety-two publications were identified from the database searches, and no relevant publications were identified by examining the study reference lists. Twenty-nine publications were excluded on account of duplication, and 44 were excluded after screening the titles and abstracts. Finally, 19 full-text articles were reviewed. Of those articles, 2 reviews, 2 commentaries, 1 case report, and 3 abstract only were excluded. Three articles about other variations of ALPPS (1 about mini-ALPPS, and 2 about ALPTIPS) and 3 articles with no comparison group were excluded. Another article was excluded because of data duplication. Finally, 4 clinical studies containing 124 patients were included in the meta-analysis.

### Characteristics of the included studies

In the 4 included studies [8–10, 13], 62 patients received partial ALPPS and 62 patients received complete ALPPS. In the study by Alvarez et al. 1 patient underwent both stages of ALPPS by a pure laparoscopic technique. All of the other patients in the 4 studies had ALPPS performed by the open approach. The basic characteristics of the included studies are summarized in Table 1. All studies were designed to compare the outcomes between partial ALPPS and complete ALPPS, and all studies were retrospective studies. Assessed by the NOS, all studies received a score of > 5 stars (Table 2).

**Table 1:** The characteristics of the included studies

Author	Year	Number of patients	Amount of parenchymal transection in partial ALPPS	FLR hypertrophy,*%*	Time interval of two stages,*d*	Postoprative complications,*n*	Hospital stay,*d*	Mortality	NOS score
Alvarez, F. A [8]	2015	*P* 21 *C* 9	_	*P* 90 *C* 107	7	*P* 8 *C* 8	_	6.60%	6
Henrik Petrowsky [9]	2015	*P* 6 *C* 18	50 to 80%	*P* 60 *C* 61	*P* 11(7–21) *C* 9(7–69)	*P* 2 *C* 6	*P* 2 *C* 3.5	*P* 0 *C* 22%	6
Chan, A [10]	2017	*P* 12 *C* 13	50 to 80%	*P* 43.1 *C* 64.1	*P* 10.5 *C* 7	*P* 3 *C* 1	*P* 22(12–40) *C* 17.5(12–25)	*P* 16.7% *C* 0	8
Linecker, M [13]	2017	*P* 23 *C* 22	61% (34–86%)	*P* 64 *C* 60	*P* 15(7–41) *C* 17(7–69)	*P* 14 *C* 20	_	*P* 0 *C* 27.3%	8

*P*:partial ALPPS group, *C*:complete ALPPS group

**Table 2:** Quality assessment using Newcastle-Ottawa Scale of included studies

Newcastle-Ottawa Scale	Studies			
	Alvarez, F. A(2015)	Henrik Petrowsky(2015)	Linecker, M(2017)	Chan, A(2017)
Selection				
Representativeness of the exposed cohort (maximum:*)	*	*	*	*
Selection of the non exposed cohort (maximum:*)	*	*	*	*
Ascertainment of exposure (maximum:*)	*	*	*	*
Demonstration that outcome of interest was not present at start of study (maximum:*)	–	–	–	–
Comparability				
Comparability of cohorts on the basis of the design or analysis (maximum:**)	–	–	**	**
Outcome				
Assessment of outcome (maximum:*)	*	*	*	*
Was follow-up long enough for outcomes to occur (maximum:*)	*	*	*	*
Adequacy of follow up of cohorts (maximum:*)	*	*	*	*
Total NOS score	******	******	********	********

The symbol "*" refers to the score of each item

### FLR hypertrophy

**Fig. 2:**
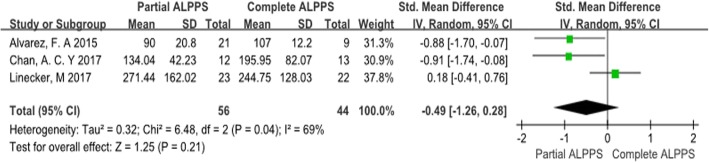
Forest plot and meta-analysis of future liver remnant regeneration

Because the study by Petrowsky et al. [9] reported no significant difference in FLR hypertrophy between partial and complete ALPPS (60% vs. 61%) without any other relevant information, only the other 3 studies were summarized in Fig. 2. SMD was used for comparison because different indexes of measurement were used in these studies. Moderate heterogeneity was observed between the studies (chi-square = 6.48; df = 2; *p* = 0.04; *I*^2^ = 69%). The results indicated that FLR hypertrophy in the partial ALPPS group and complete ALPPS group was comparable (SMD = − 0.49; 95% CI: − 1.26-0.28; *p* = 0.21).

### Time interval between stages

**Fig. 3:**
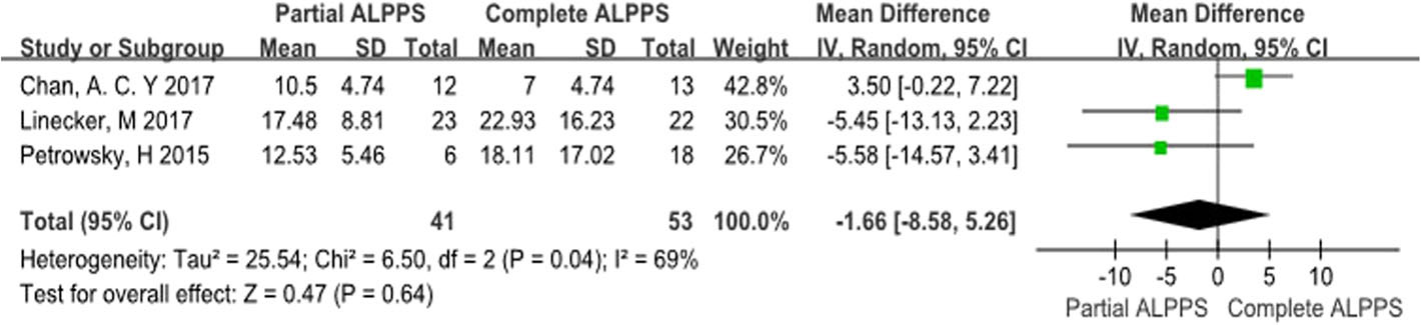
Forest plot and meta-analysis of the time interval between stages

The study by Alvarez et al. [8] only reported a total median time interval of 7 days, without range, for both groups. Thus, we only summarized the other 3 studies in Fig. 3. Moderate heterogeneity was observed between the studies (chi-square = 6.50; df = 2; *p* = 0.04; *I*^2^ = 69%). Analysis indicated there was no difference in the time interval between stages between the complete ALPPS and partial ALPPS groups (WMD = − 1.66; 95% CI: − 8.58-5.26; *p* = 0.64).

### Postoperative complications

**Fig. 4:**
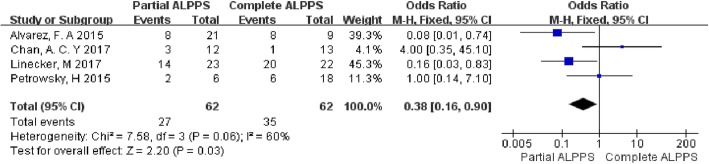
Forest plot and meta-analysis of postoperative complications

All 4 studies were included in the analysis of postoperative complications (Fig. 4). Moderate heterogeneity was observed between the studies (chi-square = 7.58; df = 3; *p* = 0.06; *I*^2^ = 60%). In partial ALPPS group, 43.5% of patients experienced postoperative complications, which was significantly lower than the 56.5% observed in the complete ALPPS group (OR = 0.38; 95% CI: 0.16–0.90; *p* = 0.03).

### Mortality rate

**Fig. 5:**
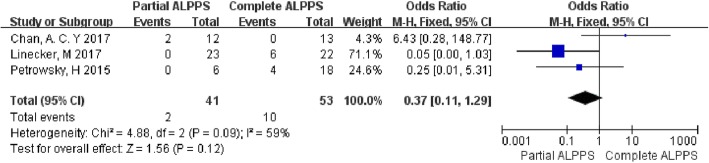
Forest plot and meta-analysis of mortality after stage 2 operation

The study by Alvarez et al. [8] only reported a total mortality rate of 6.60% for both groups. Thus, only the other 3 studies were included in the analysis (Fig. 5). Moderate heterogeneity was observed between the studies (chi-square = 4.88; df = 3; *p* = 0.09; *I*^2^ = 59%). The analysis showed a 4.9% mortality rate in the partial ALPPS group, and a 18.9% mortality rate in the complete ALPPS group; however, the difference was not statistically significant (OR = 0.37; 95% CI: 0.11–1.29; *p* = 0.12).

## Discussion

The results of this meta-analysis indicated that FLR hypertrophy and time between stages were not different between partial ALPPS and complete ALPPS. However, the postoperative complications rate in the partial ALPPS group was significantly lower than that in the complete ALPPS group, and while the mortality rate in the partial ALPPS group was markedly lower than in the complete ALPPS group the difference did not reach statistical significance.

Although ALPPS has become very popular for achieving fast hypertrophy of the FLR, the high morbidity and mortality of the procedure are important obstacles for its use. Mortality rates of 15–20% have been reported, even by experienced centers [14–17]. Alvarez et al. suggested that partial parenchymal transection could reduce morbidity without negatively impacting FLR hypertrophy [8]. Petrowsky et al. reported that partial ALPPS was associated with a zero postoperative mortality rate, and lower morbidity than complete ALLPS [9]. Based on clinical and experimental evidence, Linecker et al. reported that at least 50% of the liver needed to be transected in partial ALPPS to trigger equal volume hypertrophy as observed in complete ALPPS [13]. In the articles included in the current meta-analysis, partial ALPPS induced comparable FLR hypertrophy with a lower postoperative complications rate, consistent with prior reports. However, in 2017 Chan et al. reported an opposite result; complete ALPPS induced more rapid FLR hypertrophy than partial ALPPS while not affecting the perioperative risk [10]. Since this study was the only one that included patients with hepatocellular carcinoma (HCC), more studies are required to establish outcomes of ALLPS in patients with HCC.

The mechanism by which ALPPS induces rapid FLR hypertrophy is not clear. There are 2 main hypotheses that have been put forward to account for this phenomenon. One is that the redistribution of portal vein flow causes hypertrophy, which is based on the hypertrophy observed in patients with increased blood flow due to a portal vein embolization (PVE) [18]. Another hypothesis is that growth factors released in response to tissue injury trigger liver regeneration. Experiments using animal models strongly support the hypothesis of growth factors. Injection of plasma from ALPPS animals has been shown to induce rapid liver regeneration in animals that have not undergone ALPPS [19]. In addition, levels of interleukin (IL)-6 and tumor necrosis factor α (TNFα) are elevated after stage 1 hepatectomy in animal models and humans [19]. An interesting study by Langiewicz et al. found a clinical relevance between the Indian hedgehog (Ihh) pathway and ALPPS: liver regeneration was achieved through the hedgehog signaling pathway after both chemical injury and resection. Rapid regeneration, similar to that seen with ALPPS, was observed after administration of recombinant Ihh after PVL, while this phenomenon was blocked by neutralization of Ihh [20]. This suggests that ALPPS activates liver regeneration via the Ihh pathway.

The reason partial ALPPS is safer than complete ALLPS is likely because it is less invasive [21, 22]. Similarly, laparoscopy-assisted ALPPS [23, 24], mini ALPPS [25], and portal vein embolization ALPPS adopt less invasive operative strategies to split the diseased hemi-liver in stage 1, which enhances the tolerability of the stage 2 operation. It is important to understand that the degree of liver partitioning affects FLR hypertrophy, and determines the timing and operative strategy of stage 2. As shown in Table 1, at least 50% liver transection is needed to induce a degree of liver regeneration comparable to complete ALPPS. Time interval between the 2 stages is generally accepted as a factor that determines the surgical difficulty of stage 2; a longer time interval between stages increases the difficulty of the second stage due to greater adhesions [10].

In spite of the potential benefits, a complete ALPPS may not be applied due to difficulty of parenchymal transection in some cases. For example, when a large tumor stretching the middle hepatic vein in right anterior section, it may cause severe venous bleeding during complete ALPPS. A tumor located close to the IVC or caudate also is not feasible for complete ALPPS. In these situations, a p-ALPPS may be preferred and our study may provide essential information.

There are limitations to this study. The included studies were retrospective, and lack the powerful evidence of randomized controlled trials (RCTs), which are still absent with respect to this topic. The indications for ALLPS varied among the studies. In addition, the scarcity of available literature comparing partial and complete ALPPS and the exiguous number of patients hampered the quality of the results.

## Conclusion

The results of this meta-analysis indicated that FLR hypertrophy and time between stages were not different between partial ALPPS and complete ALPPS. However, the postoperative complications rate in the partial ALPPS group was significantly lower than that in the complete ALPPS group, and while the mortality rate in the partial ALPPS group was markedly lower than in the complete ALPPS group the difference did not reach statistical significance. More studies are needed to confirm the benefits of partial ALLPS, and indications for the procedure.

## Data Availability

The datasets used and/or analysed during the current study available from the corresponding author on reasonable request.

## Abbreviations

ALPPS: associating liver partition and portal vein ligation for staged hepatectomy
CRLM: colorectal liver metastases
FLR: future liver remnant
HCC: Hepatocellular Carcinoma
*Ihh*: Indian hedgehog
NOS: Newcastle-Ottawa Scale
OR: odds ratio
PHLF: posthepatectomy liver failure
PVE: portal vein embolization
PVL: portal vein ligation
RCT: randomized controlled trials
SMD: standard mean difference
TSH: two-stage hepatectomy
WMD: Weighted mean difference
